# Interleukin 6 trans-signalling and the risk of future cardiovascular events in men and women

**DOI:** 10.1136/openhrt-2021-001694

**Published:** 2021-10-11

**Authors:** Yasmin Miri, Karin Leander, Per Eriksson, Bruna Gigante, Louise Ziegler

**Affiliations:** 1 Division of Cardiovascular Medicine, Karolinska Institutet, Stockholm, Sweden; 2 Institute of Environmental Medicine, Karolinska Institutet, Stockholm, Sweden; 3 Department of Clinical Sciences Danderyd Hospital, Karolinska Institutet, Stockholm, Sweden

**Keywords:** biomarkers, inflammation, coronary artery disease, stroke, epidemiology

## Abstract

**Objective:**

Pro-inflammatory interleukin 6 (IL6) trans-signalling is associated with increased risk of cardiovascular events (CVEs). Whether this association exists for both sexes is, however, uncertain. Hence, we analysed the risk of CVE associated with IL6 trans-signalling in men and women and investigated if potential interaction between IL6 trans-signalling and sex affects the risk.

**Methods:**

In a prospective cohort of 60-year-old men and women without cardiovascular disease (men=2039, women=2193), subjects were followed for 20 years. To assess the IL6 trans-signalling activity, the proportion between the active binary and inactive ternary IL6 complexes, the binary/ternary ratio (B/T ratio), was estimated. CVE (myocardial infarction, angina pectoris and ischaemic stroke, n=629) risk was analysed with Cox regression, presented as HRs with 95% CIs. B/T ratio was dichotomised, with levels >median representing IL6 trans-signalling. Interaction was analysed on the additive scale and expressed as the synergy index (S). Analyses were adjusted for cardiovascular risk factors.

**Results:**

B/T ratio >median was associated with increased CVE risk in men (HR 1.63; 95% CI 1.32 to 2.01), but not in women (HR 1.21; 95% CI 0.93 to 1.57). There was a significant synergistic interaction (S=1.98; 95% CI 1.15 to 3.42) between the B/T ratio and male sex, the combination increasing the risk by 88%.

**Conclusions:**

Our results suggest differential susceptibility to inflammation mediated by IL6 trans-signalling and subsequent CVE in men and women. The B/T ratio could be considered as a novel biomarker for cardiovascular risk in men, but not in women.

**Key questions**

**What is already known about this subject?**

- Atherosclerosis is an inflammatory disease and pro-inflammatory interleukin 6 trans-signalling is associated with an increased risk of cardiovascular events. It is well known that the risk of cardiovascular disease differs between men and women. Yet, it is uncertain whether IL6 trans-signalling mediates the same effect in both sexes.

**What does this study add?**

- This prospective cohort study provides insight into the differential association between cardiovascular risk and inflammation driven by IL6 trans-signalling in men and women.

**How might this impact on clinical practice?**

- We found that men had an association between biomarkers of IL6 trans-signalling and an increased risk of CVE. The association could not be found in women. With the finding that men experience detrimental effects from IL6 trans-signalling as opposed to women, we conclude that men could possibly benefit from preventive anti-inflammatory treatment targeting IL6 trans-signalling and the IL6 trans-signalling biomarkers could thus be used to identify men suitable for treatment.

## Introduction

Sex differences in cardiovascular disease (CVD) pathophysiology, clinical presentation and prognosis are well known; for example, women display a higher prevalence of non-obstructive coronary heart disease such as stress-related cardiomyopathy, vasospasm and spontaneous coronary artery dissection.^1 2^

Inflammation plays a pivotal role in the pathophysiology of atherosclerosis. Recently, the CANTOS study showed that inhibiting interleukin (IL) 1β in the inflammatory IL1β–IL6–C reactive protein (CRP) pathway results in beneficial effects in preventing future cardiovascular events (CVEs) in patients with inflammatory activity.^3^ However, in cardiovascular clinical studies women are underrepresented.

Downstream from IL1β is IL6, a cytokine known to mediate disparate processes depending on which of its two signalling pathways is active, classic signalling or trans-signalling. In classic IL6 signalling, IL6 binds to the membrane-bound IL6 receptor (IL6R), expressed on hepatocytes, leucocytes and T cells. The signal is transduced by the binding to the ubiquitously expressed membrane-bound glycoprotein 130 (gp130).^4^ Classical IL6 signalling induces the acute-phase reaction with the production of CRP and mediates tissue homeostatic and anti-inflammatory effects.^4^ In IL6 trans-signalling, on the other hand, IL6 binds to a soluble IL6R isoform (sIL6R) forming the circulating IL6:sIL6R (binary) complex hence enabling a systemic scope of impact.^5^ IL6 trans-signalling possesses detrimental pro-inflammatory effects and is regulated by the soluble gp130 (sgp130) binding the binary complex and forming the inactive IL6:sIL6R:sgp130 (ternary) complex.^5^

Our group recently explored the risk of future CVE associated with pro-inflammatory IL6 trans-signalling using a novel biomarker consisting of a ratio between the active binary IL6:sIL6R complex and the inactive ternary IL6:sIL6R:sgp130 complex, the binary/ternary complex ratio (B/T ratio).^6^ We found that a B/T ratio >the median, mirroring a relative excess of the active binary IL6 complex, was associated with an increased risk of first-time CVE.^6^

In a study of men and women without established CVD, women had higher pro-inflammatory markers compared with men.^7^ In light of this study and clinical studies demonstrating differences in CVD risk in men and women, we hypothesised that the impact of IL6 trans-signalling on CVD risk differs between men and women.

The aim of the present study was to analyse the risk of first-time CVE associated with IL6 trans-signalling in men and women, respectively. In addition, this study aims to investigate if potential differences in risk associated with B/T ratio are dependent on the interaction between biological sex and IL6 trans-signalling.

## Materials and method

The study was designed and conducted as a prospective cohort study in accordance with the Declaration of Helsinki. All study participants were thoroughly informed about the study before giving their informed consent and entering the study.

### Study population

From the Swedish population register, every third man and woman turning 60 and living in the Stockholm County between 1 July 1997 and 30 June 1998 were randomly selected and invited to participate in a cardiovascular health screening study. A total of 4232 subjects (2039 men and 2193 women) replied positively (78%) and were included. A questionnaire with information regarding lifestyle, current and previous diseases, and medication was filled out by the participants. All subjects underwent a thorough physical examination, an ECG and blood sampling. The cohort is described in detail elsewhere.^6^

### Biochemical analyses

Fasting blood samples were collected at baseline and stored in −80°C in a biobank. From the collected samples, serum levels of IL6 and sIL6R were analysed with Mesoscale Discovery Systems Cytokine Assay (Gaithersburg, MD, USA) and sgp130 with an assay development kit (#DY228) from R&D Systems (R&D Systems, Minneapolis, MN, USA). All experiments were performed according to the manufacturer’s protocol. Descriptions of the experimental procedures have been previously published.^6^ Concentrations of IL6 were expressed in picograms per millilitre (pg/mL) and sIL6R and sgp130 in nanograms per millilitre (ng/mL). Owing to the fact that IL6, sIL6R and sgp130 interact on a molar level, the molar concentrations (moles per litre) of the binary (IL6:sIL6R) and the ternary (IL6:sIL6R:sgp130) complex, both expressed in nanomoles per litre, were estimated. Formulas presented by Garbers and Müller-Newen were used for this purpose and the calculations have been described in detail in prior publications.^6 8 9^

### Outcome

The personal identification numbers of the subjects were linked to the national Swedish registers, the Hospital Discharge Register and the National Cause of Death Register, to extract diagnoses. Main diagnoses were recorded until 31 December 2017. The outcome was first-time fatal or non-fatal CVE with the following diagnosis codes from the International Classification of Diseases 10th revision: myocardial infarction (MI) (I21), coronary heart disease (I20 and I25), sudden cardiac death (I46) and ischaemic stroke (I63). After excluding subjects with incomplete questionnaires (n=122), lacking serum samples (n=96) and those with prevalent CVD (n=369, men=225, women=144), there were 654 incident CVE cases. Additional 26 subjects were restricted from analyses due to inaccurately having been classified as cases, leaving 3619 subjects with 629 cases of fatal and non-fatal CVE (MI n=221, hospitalised angina pectoris n=202, cardiac arrest n=3 and ischaemic stroke n=203) in the final analysis. For a detailed description of the included/excluded study participants, please see online supplemental figure 1.

10.1136/openhrt-2021-001694.supp1 Supplementary data

### Statistical analysis

Continuous variables are presented as median and IQR. Binary variables are presented as percentages.

The relative risk of first-time CVE associated with IL6, sIL6R, sgp130 and the B/T ratio was estimated using Cox proportional hazards model and expressed as HR with 95% CIs. The significance level was set at 5%. All analyses were performed in men and women separately.

In initial analyses, the association between each component of the binary (IL6:sIL6R) and ternary (IL6:sIL6R:sgp130) complex and the outcome was analysed. Each component was analysed both as a continuous variable and categorised into quartiles. The quartile boundaries for the individual components in each sex can be found in online supplemental table 1.

The risk of CVE associated with the B/T ratio was first analysed with the B/T ratio as a continuous variable with 0.1 unit increase due to the narrow range of the variable (1.29–2.29) and in additional analyses the B/T ratio was categorised into quartiles. Based on the results from these analyses, the B/T ratio was dichotomised at the median for men (1.59) and women (1.58), respectively, and the association between the B/T ratio >median, mirroring a relative excess of the active binary complex in relation to the inactive ternary complex and CVE risk, was analysed with the reference group being B/T ratio ≤median.

The analysis of potential interaction between the two dichotomised exposures, sex (female vs male) and IL6 trans-signalling (B/T ratio ≤ vs >median), was made on the additive scale. The four groups of exposures were as follows: women with B/T ratio ≤median (reference group); women with B/T ratio >median; men with B/T ratio ≤median; men with B/T ratio >median. To assess interaction on an additive scale, the synergy index (S) is presented with S=1 indicating an absence of interaction, S >1 suggesting a synergistic effect and S <1 an antagonistic effect between the exposures. S is presented with 95% CI and p value with a significance level set at 5%. An extended interaction analysis, including the relative excess risk due to interaction (RERI) and attributable portion (AP), is presented in online supplemental material.

All analyses are presented in univariate and multivariate models. In the latter, hypertension was defined as blood pressure >140/90 mmHg and/or medication for hypertension and/or self-reported in the questionnaire, diabetes mellitus as fasting glucose >7.0 mmol/L and/or treatment for diabetes mellitus and/or self-reported, hyperlipidaemia as fasting total serum cholesterol >5.0 mmol/L and/or treatment for hyperlipidaemia and/or self-reported. In addition, the use of menopausal hormone therapy (MHT) by female post-menopausal participants was self-reported in the questionnaire.

A complete-case analysis was performed to analyse the presence of potential effects of missing data on the results. No indication of such influence was found (data not shown).

All analyses were performed using StataCorp (Stata Statistical Software: Release 14. College Station, TX: StataCorp LP).

### Patient and public involvement

This study is a population-based cohort study where every third 60-year-old man and woman living in Stockholm County was invited to participate in a cardiovascular health screening study.

## Results

The clinical and biochemical baseline characteristics of the study population are shown in table 1. Men were overrepresented in the group of participants who experienced a CVE during follow-up. Men also had a higher prevalence of hyperlipidaemia and diabetes mellitus. IL6, sIL6R and sgp130 were all measured in higher concentrations in men compared with women. The B/T ratio level was, however, similar between the sexes.

**Table 1:** Baseline characteristics of the study population

	All	Men	Women
Subjects, n (%)	3619	1705 (47)	1914 (53)
CVE, n (%)	629	396 (63)	233 (37)
Anthropometric data			
Body mass index (kg/m^2^)	26.2 (23.8–28.9)	26.5 (24.3–28.9)	25.9 (23.8–28.9)
Systolic pressure (mm Hg)	136 (122–152)	141 (128–155)	132 (118–148)
Diastolic pressure (mm Hg)	84 (77–91)	87 (80–94)	81 (75–88)
Biochemical values (mmol/L)			
Total cholesterol	5.9 (5.3–6.6)	5.8 (5.1–6.5)	6.1 (5.4–6.7)
LDL	3.8 (3.2–4.5)	3.8 (3.2–4.4)	3.8 (3.2–4.5)
HDL	1.5 (1.2–1.7)	1.3 (1.1–1.5)	1.6 (1.4–1.9)
Fasting glucose	5.2 (4.8–5.7)	5.3 (5.0–5.8)	5.1 (4.7–5.5)
Cardiovascular risk factors, %			
Hypertension	15.8	15.8	15.7
Hyperlipidaemia	3.5	4.2	2.9
Diabetes mellitus	6.1	8.5	4.1
Current smoking	21	20	22
Ongoing medical treatment, %			
Menopausal hormone therapy	NA	NA	9.0
IL6 system biomarkers			
IL6 (pg/mL)	0.59 (0.42–0.89)	0.62 (0.44–0.90)	0.56 (0.39–0.88)
sIL6R (ng/mL)	33.4 (27.1–41.5)	35.8 (29.0–44.4)	31.4 (25.7–38.4)
sgp130 (ng/mL)	383 (320–451)	396 (339–459)	368 (306–443)
Binary/ternary complex ratio			
B/T ratio	1.59 (1.55–1.62)	1.59 (1.56–1.62)	1.58 (1.54–1.61)

Continuous variables are presented as median (IQR) and categorical variables are presented in percentages. CVE n, (%)=number of CVEs and the proportion of CVEs in the cohort and each subgroup. Missing values (men/women): systolic and diastolic blood pressure n=3 (2/1), LDL n=45 (34/11), current smoking n=44 (20/24).

CVE, cardiovascular event; HDL, high-density lipoprotein; LDL, low-density lipoprotein; NA, not applicable.

### IL6, sIL6R, sgp130 and the risk of future CVE

There was a linear association between IL6 and sIL6R, respectively, and the risk of future CVE in men as seen in online supplemental table 2. A pattern of linear association with the outcome could be discerned for IL6 and sIL6R also in women although without statistical certainty (online supplemental table 3). The association between sgp130 and the outcome was not significant in either sex although the association analysis suggests a non-linear pattern (online supplemental tables 2 and 3).

### B/T ratio and the risk of future CVE

In men, each 0.1 unit increase of the B/T ratio was associated with an increased risk (adjusted HR 1.58; 95% CI 1.27 to 1.97) as was each B/T ratio quartile increase (adjusted HR 1.20; 95% CI 1.10 to 1.31), please see online supplemental table 4. When analysing the risk associated with each quartile of the B/T ratio, with the lowest quartile as the reference group, a significant risk increase was seen for B/T ratio levels above the median (online supplemental table 2). In table 2, the B/T ratio was dichotomised at the median and levels >median were associated with a 58% risk increase.

**Table 2:** B/T ratio and risk of future cardiovascular events

	Crude HR (95% CI)	P value	Adjusted HR (95% CI)	P value
Men				
B/T ratio >median	1.69 (1.38 to 2.07)	<0.001	1.58 (1.29 to 1.94)	<0.001
Women				
B/T ratio >median	1.34 (1.03 to 1.73)	0.03	1.29 (1.00 to 1.68)	0.05

Risk of CVE associated with the B/T ratio expressed as HR with 95% CI. The reference group was B/T ratio ≤median. The analysis is stratified by sex and adjusted for diabetes, hypertension, hyperlipidaemia, BMI and smoking. For women, further adjustments were made for MHT.

BMI, body mass index; CVE, cardiovascular event; MHT, menopausal hormone therapy.

In women, results from the same analyses demonstrated smaller risk estimates without statistical significance, although the pattern of association was similar to that in men (table 2, online supplemental tables 3 and 4).

### Interaction between sex and IL6 trans-signalling on the risk of CVE

**Figure 1:**
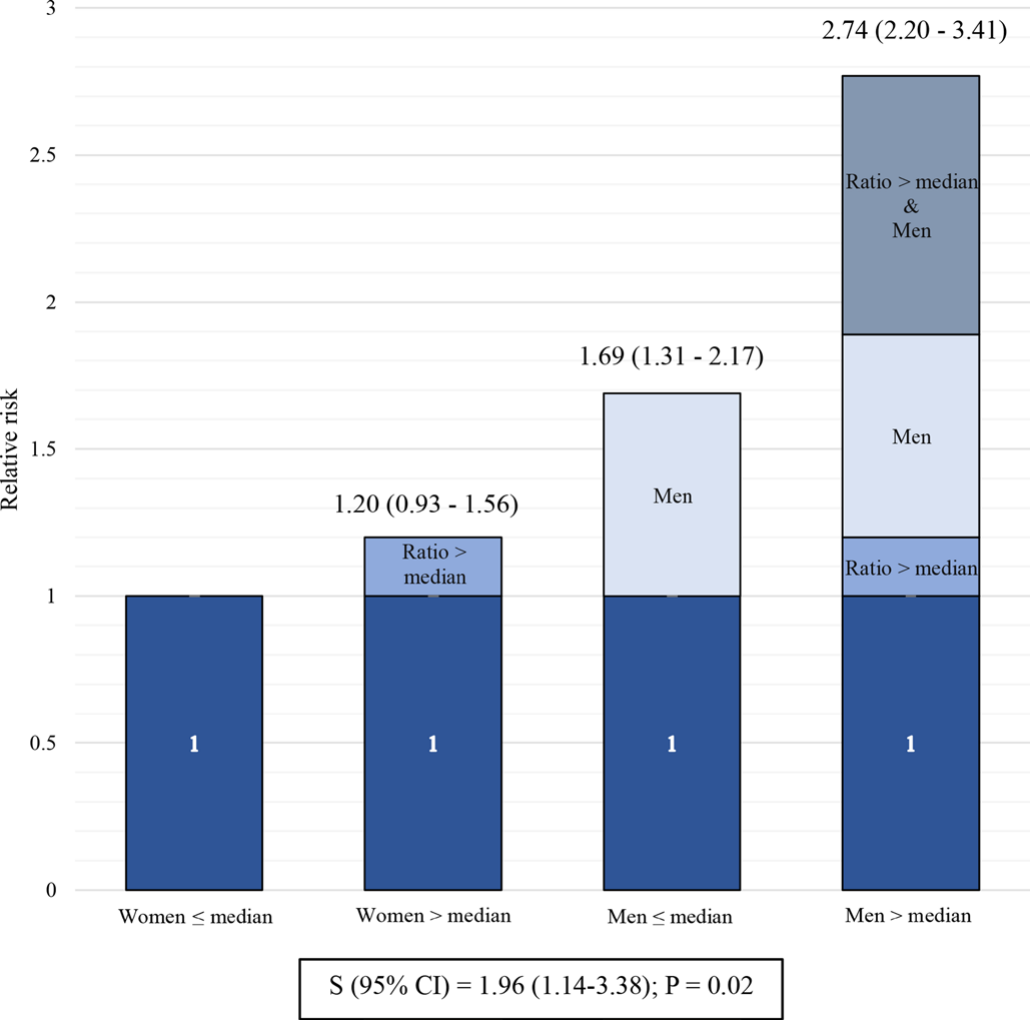
Interaction analysis of combined exposure of biological sex and IL6 trans-signalling. The bars graphically represent the relative risk of cardiovascular event (HR; 95% CI) associated with different combinations of exposures of biological sex and IL6 trans-signalling. The reference group is the combination of female sex and B/T ratio ≤median (HR 1.0). The measure of additive interaction, synergy index (S), is presented with 95% CI. The analysis is adjusted for diabetes, hypertension, hyperlipidaemia, body mass index and smoking.

To evaluate whether biological sex potentially modified the association between IL6 trans-signalling and the risk of CVE, the risk in different exposure groups was estimated. The group with lowest expected risk, female sex and B/T ratio ≤median, was used as the reference. As shown in figure 1, when adding the exposure of B/T ratio >median to female sex there was a small and statistically insignificant risk increase. Instead, the combination of male sex and B/T ratio ≤median was associated with a significant risk increase. The highest risk was seen for the combination of male sex and B/T ratio >median and the risk estimate in this group was greater than the sum of the individual risks together, indicating an interaction between the two exposures. Synergy index >1 confirmed an interaction between male sex and IL6 trans-signalling on the risk of CVE.

## Discussion

This is the first study exploring the association of pro-inflammatory IL6 trans-signalling, mirrored by the B/T ratio, with the risk of future CVE in men and women, respectively. The main finding is that IL6 trans-signalling is associated with an increased risk of CVE in men but not in women. In addition, there is an additive interaction between the biological sex and IL6 trans-signalling on the CVE risk.

The IL6 pathway is involved in atherosclerosis-related CVD with elevated IL6 concentrations being associated with atherosclerotic plaque formation,^10^ microvascular dysfunction^11^ and an increased risk of MI.^12^ High levels of circulating sIL6R are associated with an increased risk of CVD^13^ and adverse outcomes in patients with MI,^14 15^ and is causally related to CVD in Mendelian randomisation studies.^16 17^ In line with this, we show a linear association for IL6 and sIL6R with the outcome primarily in men.

Studies regarding the effects of the IL6 trans-signalling inhibitor, sgp130, are inconsistent. High concentrations of sgp130 have been associated with a decreased risk of future CVE^13 18^ but also with a negative prognosis in coronary heart disease.^14 19^ We previously showed a non-linear association for sgp130 with CVE in unstratified analyses of the cohort of 60-year-olds with extremely high and low concentrations being associated with a lower CVE risk.^6^ Potentially elevated sgp130 levels mirror an activated IL6 trans-signalling buffer whereas low values represent a low activity of IL6 trans-signalling. In the present analysis, we found an indication of a similar non-linear association pattern in women.

Because IL6, sIL6R and sgp130 form the active binary and inactive ternary IL6 complexes on a molar level, the impact of the IL6 trans-signalling pathway cannot be evaluated with IL6 or the soluble receptors individually. We therefore constructed the B/T ratio, a combined IL6 trans-signalling marker. In the cohort of 60-year-olds, B/T ratio levels >median, mirroring active signalling, are associated with an increased risk of a first-time CVE^6^ and provide prognostic information on the risk of future CVE for individuals defined as having a low–intermediate cardiovascular risk.^20^

In the present study, we show that B/T ratio >median is associated with an increased risk of future CVE in men but not in women.

In an Asian coronary artery disease case–control study of postmenopausal women, on the other hand, the B/T ratio was significantly higher in cases compared with controls although an association could not be demonstrated.^18^ These findings together with ours indicate that the B/T ratio may not be an appropriate predictive cardiovascular biomarker in women. Of note, the Asian study included women with acute coronary disease and thus explored the B/T ratio as a diagnostic biomarker. Results from this study are hence not completely comparable with those in ours.

One possible explanation for the differentiating results in men and women could be sex differences related to the pathophysiology of the culprit plaque. Recent studies have shown that plaque erosion with overlying thrombosis display a lower inflammatory activity and infiltration of inflammatory cells compared with ruptured plaques.^21^ However, in modern studies, no difference in frequency of erosions between men and women have been demonstrated.^21^

The results of the interaction analysis suggest that a substantial proportion of the increased CVE risk can be attributed to the interaction between male sex and IL6 trans-signalling. One can speculate that female sex is protective of the detrimental IL6 trans-signalling effects in line with the favourable effects in systemic inflammation–induced endothelial dysfunction seen in women.^22–24^ The protective mechanisms are likely multifactorial, involving both genetic and hormonal factors. Several inflammatory proteins, some part of the IL1β pathway, are encoded on the X-chromosome.^25^ Compared with men, lower IL6 levels are seen in women and men with Klinefelter syndrome, both having two X-chromosomes.^24^ In light of this, it can be speculated that the male sex constitutes a more pro-inflammatory phenotype, with a greater susceptibility to IL6 trans-signalling and its consequences.

### Strengths and limitations

This study has several limitations. Primarily, it is an observational study and thus we cannot draw any conclusions on mechanisms. Furthermore, the statistical analyses are data driven although our goal is not to establish appropriate cut-offs for the B/T ratio but merely explore potential associations. Moreover, the inflammatory biomarkers were measured at baseline and we cannot exclude possible changes in concentrations over time. Also, we have not measured concentrations of sex hormones known to influence the development of atherosclerosis.^26^ In addition, some of the coronary events in the cohort could be due to non-obstructive coronary disease entailing potentially differential pathophysiology and more common in women hence possibly introducing misclassification bias.^1^ We do, however, not have the information on the presence of coronary plaques or plaque morphology in this study. The proportion of coronary events due to non-obstructive coronary atherosclerosis in epidemiological studies is, however, low and a large proportion of these have been shown to be caused by atherosclerosis.^1^ Moreover, high concentrations of IL6 have been seen in women with Takotsubo.^27^ In addition, since we excluded participants with prevalent CVD, the analysed group of women had lower prevalence of risk factors possibly leading to a lower incidence rate of CVE, thus resulting in reduced power to detect an association. Furthermore, we have not analysed the association with cardiovascular mortality but merely analysed the risk of non-fatal and fatal CVE without having enough power to perform separate analyses of the two.

The main strength of this study is that it is a large prospective population-based cohort with nearly complete 20-year follow-up. Hence, the results should be generalisable to similar populations. In addition, we have included all three components that constitute and regulate IL6 trans-signalling in one biomarker.

### Clinical application/future perspectives

Lately, clinical trials have demonstrated the preventive effect of anti-inflammatory treatment by inhibiting IL1β and IL6 in patients with established CVD.^3 28 29^ In the IL1β–IL6–CRP pathway, only IL6 signalling has been shown to be causally associated with CVD.^16 17^ Moreover, recombinant sgp130 has atherosclerosis dampening properties demonstrated in experimental studies.^30^ Hence, targeting the pro-inflammatory IL6 trans-signalling pathway is of great interest.

Our findings suggest that men experience negative cardiovascular effects when exposed to IL6 trans-signalling and could hence benefit from anti-inflammatory treatment, for example, with recombinant sgp130. In addition, the B/T ratio could be used as a novel biomarker to identify men with an increased cardiovascular risk and monitor treatment effects. The B/T ratio has been seen in higher levels in women with ongoing CVE,^18^ although considering the lack of association in our study it is uncertain if women would benefit from treatment targeting IL6 trans-signalling.

## Conclusions

In conclusion, IL6 trans-signalling, mirrored by the B/T ratio, is associated with an increased risk of first-time CVE in men without prevalent CVD. Accordingly, the B/T ratio could be a potential biomarker for CVE prediction and identify male individuals suitable for preventive anti-inflammatory treatment. An association could, however, not be demonstrated in women. Our results suggest differential susceptibility to chronic inflammation and subsequent CVE mediated by IL6 trans-signalling in men and women.

## Data Availability

Data are available on reasonable request. Data are stored at Karolinska Institutet. Due to legal restrictions imposed by the Swedish Secrecy Act, requests for access individual data can be sent to BG (bruna.gigante@ki.se).
