# Supplementary material for: Interleukin 6 trans-signalling and the risk of future cardiovascular events in men and women
Source: Open Heart. 2021 Oct 11;8(2):e001694. doi: 10.1136/openhrt-2021-001694 (PMC8506880; doi:10.1136/openhrt-2021-001694)
Supplement: Supplementary data [file openhrt-2021-001694supp001.pdf]

Supplemental material

Interleukin 6 trans-signalling and the risk of future cardiovascular events in men and women

Yasmin Miri, MD [1]; Karin Leander, PhD [2]; Per Eriksson [1]; Bruna Gigante, MD, PhD [1,3] and Louise Ziegler, MD, PhD [4,5]

[1] Division of Cardiovascular Medicine, Danderyd Hospital, Stockholm, Sweden; [2] Unit of Cardiovascular and Nutritional Epidemiology, Institute of Environmental Medicine, Karolinska Institutet, Stockholm, Sweden; [3] Cardiovascular Medicine Unit, Department of Medicine, Karolinska Institutet, Stockholm, Sweden; [4] Dept. of Clinical Sciences, Danderyd Hospital, Karolinska Institutet, Stockholm, Sweden; [5] Division of Internal Medicine, Danderyd Hospital, Stockholm, Sweden.

Corresponding author contact information:
Louise Ziegler, MD, PhD

Karolinska Institutet, Department of Clinical Sciences

Danderyd Hospital S-182 88 Stockholm, Sweden

Tel: +46 8 123 580 48/550 00, Fax: +46 8 524 800 00

E-mail: louise.dencker-ziegler@sll.se

<u>Content:</u>

Supplemental Method

Supplemental Figure I

Supplemental Table I

Supplemental Results

Supplemental Table II-V

Supplemental Method

Supplemental figure I. Flow chart of exclusions

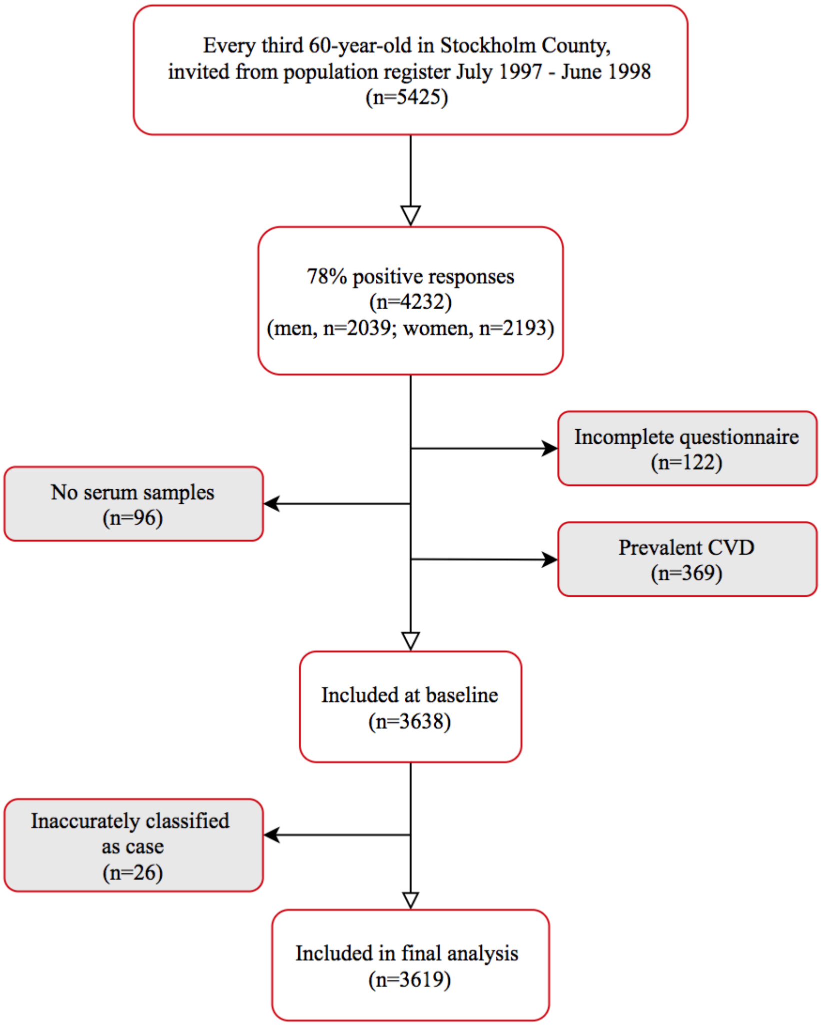

Supplemental Figure I. Subjects were excluded from the present analyses if they had incomplete questionnaires at enrolment, missing serum samples or if they had prevalent CVD at baseline; defined as self-reported or registered ICD-10 diagnosis codes of either MI (I121), chronic ischemic heart disease (I25) or ischemic stroke (I63). Furthermore, subjects who inaccurately had been categorised as cases with the following diagnosis codes were restricted from the final analysis: carotid artery stenosis (I652) if it was unclear whether the subject had a stroke or not; chronic ischemic heart disease (I259) because former events could not be excluded; old MI (I252) because the time of the event was unknown; and stroke not specified as haemorrhage or infarction (I649) because the underlying cause of the event was not clear.

Quartile boundaries for individual biomarkers

Supplemental Table I. Quartile boundaries for IL6 trans-signalling biomarkers in men and women

	≤25th	>25-≤50th	>50-≤75th	>75th
Men				
**IL6** (pg/ml)	≤ 0.44	>0.44 ≤ 0.62	>0.62 ≤ 0.90	> 0.90
**sIL6R** (ng/ml)	≤ 28.98	> 28.98 ≤ 35.76	> 35.76 ≤ 44.36	> 44.36
**sgp130** (ng/ml)	≤ 338.4	> 338.4 ≤ 395.7	> 395.7 ≤ 459.1	> 459.1
**B/T ratio**	≤ 1.56	>1.56 ≤1.59	>1.59 ≤1.62	>1.62
Women				
**IL6** (pg/ml)	≤ 0.39	>0.39 ≤ 0.56	> 0.56 ≤ 0.88	> 0.88
**sIL6R** (ng/ml)	≤ 25.68	> 25.68 ≤ 31.41	> 31.41 ≤ 38.43	> 38.43
**sgp130** (ng/ml)	≤ 306.4	> 306.4 ≤ 367.6	> 367.6 ≤ 443.2	> 443.2
**B/T ratio**	≤ 1.54	>1.54 ≤1.58	>1.58 ≤1.61	>1.61

Supplemental Table I. Quartile boundaries for each IL6 trans-signalling biomarker in men and women, respectively

Supplemental results

IL6, sIL6R, sgp130 and the risk of future CVE

Supplemental Table II: IL6, sIL6R, sgp130 and B/T ratio and risk of CVE in men

	Crude	P	Adjusted	P
**IL6**				
≤25th perc	1.00 (ref)	-	1.00 (ref)	-
25-50th perc	1.27 (0.95-1.70)	0.11	1.19 (0.89-1.60)	0.24
50-75th perc	1.19 (0.89-1.60)	0.24	1.03 (0.76-1.39)	0.85
>75th perc	1.66 (1.26-2.20)	<0.001	1.35 (1.00-1.81)	0.05
**sIL6R**				
≤25th perc	1.00 (ref)	-	1.00 (ref)	-
25-50th perc	1.21 (0.93-1.66)	0.24	1.21 (0.89-1.66)	0.22
50-75th perc	1.73 (1.30-2.32)	<0.001	1.69 (1.26-2.27)	<0.001
>75th perc	1.78 (1.33-2.38)	<0.001	1.61 (1.20-2.15)	0.002
**sgp130**				
≤25th perc	1.00 (ref)	-	1.00 (ref)	-
25-50th perc	1.24 (0.84-1.57)	0.14	1.20 (0.90-1.61)	0.21
50-75th perc	1.21 (0.91-1.61)	0.20	1.19 (0.89-1.60)	0.23
>75th	1.32 (0.99-1.75	0.06	1.21 (0.91-1.62)	0.19
**B/T ratio**				
≤25th perc	1.00 (ref)	-	1.00 (ref)	-
25-50th perc	0.98 (0.72-1.34)	0.91	1.0 (0.73-1.37)	0.99
50-75th perc	1.63 (1.22-2.17)	0.001	1.58 (1.19-2.11)	0.002
>75th	1.72 (1.30-2.29)	<0.001	1.58 (1.19-2.10)	0.002

Supplemental Table II: Risk of CVE, expressed as hazard ratio (HR) with 95% confidence interval (CI) associated with the individual biomarkers involved in IL6 trans-signalling in men. Reference group: ≤25th percentile. The analysis is adjusted for diabetes, hypertension, hypercholesterolemia, BMI and smoking.

Supplemental Table III: IL6, sIL6R, sgp130 and B/T ratio and risk of CVE in women

	Crude	P	Adjusted	P
**IL6**				
≤25th perc	1.00 (ref)	-	1.00 (ref)	-
25-50th perc	0.93 (0.63-1.38)	0.72	0.89 (0.60-1.32)	0.55
50-75th perc	1.27 (0.88-1.84)	0.20	1.07 (0.73-1.56)	0.74
>75th perc	1.48 (1.03-2.11)	0.03	1.15 (0.79-1.69)	0.46
**sIL6R**				
≤25th perc	1.00 (ref)	-	1.00 (ref)	-
25-50th perc	0.97 (0.66-1.44)	0.89	0.92 (0.62-1.37)	0.70
50-75th perc	1.15 (0.79-1.68)	0.46	1.06 (0.73-1.55)	0.75
>75th perc	1.52 (1.07-2.17)	0.02	1.34 (0.95-1.95)	0.10
**sgp130**				
≤25th perc	1.00 (ref)	-	1.00 (ref)	-
25-50th perc	1.41 (0.97-2.05)	0.08	1.42 (0.97-2.06)	0.07
50-75th perc	1.45 (1.0-2.11)	0.05	1.36 (0.93-1.98)	0.11
>75th	1.22 (0.83-1.80)	0.32	1.13 (0.76-1.68)	0.53
**B/T ratio**				
≤25th perc	1.00 (ref)	-	1.00 (ref)	-
25-50th perc	1.03 (0.69-1.51)	0.90	0.97 (0.65-1.43)	0.87
50-75th perc	1.40 (0.97-2.01)	0.07	1.30 (0.90-1.88)	0.16
>75th perc	1.31 (0.91-1.90)	0.15	1.16 (0.80-1.69)	0.42

Supplemental Table III: Risk of CVE, expressed as hazard ratio (HR) with 95% confidence interval (CI) associated with the individual biomarkers involved in IL6 trans-signalling in women. Reference group: ≤25th percentile. The analysis is adjusted for diabetes, hypertension, hypercholesterolemia, BMI, smoking and hormone replacement therapy.

B/T ratio as a continuous variable and the risk of future CVE

Supplemental Table IV. B/T ratio and the risk of CVE in men and women

B/T ratio	Crude	P	Adjusted	P
**Men**				
0.1 unit	1.71 (1.37-2.13)	<0.001	1.58 (1.27-1.97)	<0.001
Quartile	1.24 (1.13-1.35	<0.001	1.20 (1.10-1.31)	<0.001
**Women**				
0.1 unit	1.19 (0.97-1.47)	0.10	1.11 (0.90-1.38)	0.32
Quartile	1.12 (1.0-1.25)	0.06	1.08 (0.96-1.21)	0.21

Supplemental Table IV: The risk of future cardiovascular events (CVE), expressed as hazard ratio (95% confidence interval), associated with the B/T ratio per 0.1 unit and quartile increase, respectively. The analysis is performed in men and women, separately and adjusted for diabetes, hypertension, hypercholesterolemia, BMI and smoking. Further adjustments were made for hormone replacement therapy in women.

Interaction analysis

Supplemental Table V: Interaction analysis of the risk of future CVE associated with combined exposures of sex and IL6 trans-signalling

	Women		Men	
	N with/without CVE	RR (95% CI); P	N with/without CVE	RR (95% CI); P
**B/T ≤median**	117/952	1.0	130/610	1.69 (1.31-2.17); P<0.001
**B/T >median**	116/729	1.20 (0.93-1.56); P= 0.16	267/698	2.74 (2.20-3.41); P<0.001

RERI (95% CI) = 0.85 (0.36 - 1.35); P<0.001

AP (95% CI) = 0.31 (0.14-0.48); P<0.001

Supplemental Table V. Risk of CVE, expressed as hazard ratio (HR) with 95% confidence interval (CI), for combined exposures of sex and the B/T ratio. Reference group: women; B/T ratio ≤median (HR=1.0). HR, relative excess risk due to interaction (RERI) and attributable proportion (AP) are adjusted for diabetes, hypertension, hypercholesterolemia, BMI, smoking and hormone replacement therapy (in women).
